# S103. IS CANNABIS A RISK FACTOR FOR SUICIDE ATTEMPTS IN MEN AND WOMEN WITH PSYCHOTIC ILLNESS?

**DOI:** 10.1093/schbul/sby018.890

**Published:** 2018-04-01

**Authors:** Anna Waterreus, Patricia Di Prinzio, Johanna Badcock, Mat Martin-Iverson, Assen Jablensky, Vera Morgan

**Affiliations:** University of Western Australia

## Abstract

**Background:**

A growing body of evidence supports the association between cannabis use and an increased risk of suicidal behaviour in the general population. However, studies that have examined the relationship between cannabis use and suicide in people with a psychotic disorder report divergent findings. Further research is needed to help clarify the relationship between cannabis use and suicidal behaviour in men and women with psychotic illness.

**Aim:**

To examine whether past-year cannabis use by men and women with psychotic disorders was associated with an increased risk of suicide attempt, and what others factors were associated with suicide attempt, stratified by sex.

**Methods:**

Data from 1065 men and 725 women interviewed in the second Australian national survey of psychosis (SHIP) were analysed using multiple logistic regression to model separately, for each sex, the impact of daily, casual or no past-year cannabis use and other risk factors, on a past 12-month suicide attempt.

**Results:**

In the 12 months prior to interview, 168 (9.4%) participants attempted suicide. Almost one quarter (23.1%) of women using cannabis on a daily basis attempted suicide compared to 15.2% of casual users and 10.2% of non-users. In contrast, the proportion of men attempting suicide across daily, casual and cannabis non-users was 10.8%, 9.1%, and 6.1% respectively. Unadjusted analyses showed daily cannabis users of both sexes had significantly increased odds of attempting suicide compared to non-users (men OR: 1.85, 95% CI: 1.02–3.35, women OR: 2.64, 95% CI: 1.39–5.00). This relationship remained, but was no longer significant, after adjusting for other covariates. Other factors associated with a significantly increased odds of a suicide attempt were: feeling isolated and lonely for men, and homelessness and hallucinations for women. Depression had the strongest association with attempting suicide for both sexes. Analysis examining whether the influence of cannabis use on suicide attempt differed according to age group (18–34 years or 35–64 years) indicated daily cannabis was associated with higher odds of attempting suicide in older men compared to non-users (OR: 2.80 95% CI: 1.10–7.13); this was not found in younger men or women.

**Discussion:**

This study highlights the high rates of suicide attempt in people with psychotic illness (9.4% in contrast to 2.4% for the Australian general population), the increased risk of a suicide attempt associated with cannabis use, particularly for older men, and how risk factors differ between men and women. However, it also raises a number of questions regarding what are the possible mechanisms underpinning a relationship between cannabis use and suicidal behaviour, in particular, whether cannabis use has an influence on specific biological pathways, which may also explain the observed differences between men and women. With a number of countries considering legalising cannabis use, it is important for researchers to continue to clarify what impact cannabis use has on people with psychotic illness.

